# Dynamic tracking of functional gene modules in treated juvenile idiopathic arthritis

**DOI:** 10.1186/s13073-015-0227-2

**Published:** 2015-10-24

**Authors:** Nan Du, Kaiyu Jiang, Ashley D. Sawle, Mark Barton Frank, Carol A. Wallace, Aidong Zhang, James N. Jarvis

**Affiliations:** Department of Computer Sciences and Engineering, University at Buffalo, Buffalo, NY USA; Department of Pediatrics, Rheumatology Research, University at Buffalo School of Medicine, Buffalo, NY USA; The Herbert Irving Comprehensive Cancer Center, Columbia University Medical Center, New York, NY 10032 USA; Oklahoma Medical Research Foundation, Clinical Immunology Program, Oklahoma City, OK USA; Department of Pediatrics, University of Washington, Seattle, WA USA; Genetics, Genomics, and Bioinformatics Program, University at Buffalo, Buffalo, NY USA; Pediatric Rheumatology Research, University at Buffalo Clinical & Translational Research Center, 875 Ellicott St, Buffalo, NY 14203 USA

## Abstract

**Background:**

We have previously shown that childhood-onset rheumatic diseases show aberrant patterns of gene expression that reflect pathology-associated co-expression networks. In this study, we used novel computational approaches to examine how disease-associated networks are altered in one of the most common rheumatic diseases of childhood, juvenile idiopathic arthritis (JIA).

**Methods:**

Using whole blood gene expression profiles derived from children in a pediatric rheumatology clinical trial, we used a network approach to understanding the impact of therapy and the underlying biology of response/non-response to therapy.

**Results:**

We demonstrate that therapy for JIA is associated with extensive re-ordering of gene expression networks, even in children who respond inadequately to therapy. Furthermore, we observe distinct differences in the evolution of specific network properties when we compare children who have been treated successfully with those who have inadequate treatment response.

**Conclusions:**

Despite the inherent noisiness of whole blood gene expression data, our findings demonstrate how therapeutic response might be mapped and understood in pathologically informative cells in a broad range of human inflammatory diseases.

## Background

While they are typically described and studied discretely and in isolation, the multiple components of a cell (genes, proteins, metabolites, RNA molecules and their splice variants, and so on) are highly inter-connected and interactive. One of the most interesting recent discoveries in modern biology, and one that has significant implications for the understanding of human disease, is the fact that the hundreds of thousands of individual cellular components can be described and visualized as interactive networks (for example, [1–4]). Furthermore, these networks share structural characteristics that frequently include ‘scale-free’ hub and node structures [5, 6] and specific, functionally related modules [7–9]. We [10] and others [11, 12] have proposed that human illnesses emerge as a consequence of perturbation of these networks, whether from genetic variation, direct external stimuli (for example, toxins, infectious agents), or via epigenetic changes that accumulate over generations; these three categories, of course, are not mutually exclusive. There is ample evidence for this viewpoint in model organisms; physiologic perturbation of yeast, for example, results in extensive remodeling of interaction networks in such a way that the vast majority of interactions seen in the resting state are no longer seen after perturbation [13].

Juvenile idiopathic arthritis (JIA) is a complex trait characterized by known genetic susceptibility [14] and presumed gene-environment interactions [15]. The hallmark pathology of JIA is the presence of inflamed and hypertrophied synovium in one or more joints, characteristically accompanied by morning stiffness and limited range of motion [16]. The illnesses classified under the nosologic entity ‘JIA’ have several different categories, each of which is considered to be distinct both phenotypically and immunogenetically. Two of the major categories, polyarticular JIA (rheumatoid factor negative and rheumatoid factor positive), resemble adult rheumatoid arthritis [17]. As with adult rheumatoid disease, the causes(s) of polyarticular JIA are unknown and therapy remains largely empiric. However, effective agents are available and prolonged periods of normal function without disease activity are now possible for many children with this disease [18].

Previous work by our group has demonstrated the presence of complex gene co-expression networks in JIA and other pediatric rheumatic diseases [10]. These networks involve cells of both the innate [19] and adaptive [20] immune systems. More recently, Stevens *et al.* [21] used genetic association and publicly available gene expression data to elucidate complex network structures in JIA. However, these analyses, including our own, have not attempted to examine the complex, dynamic changes to network properties and structure that likely underlie disease progression or therapeutic response.

The Trial of Early Aggressive Therapy in JIA (TREAT) study represents a once-in-a-generation opportunity to observe therapeutic response in polyarticular JIA in a controlled setting using agents of known efficacy. The TREAT study was an NIH-funded clinical trial [22] that compared two aggressive therapeutic regimens for treatment of newly diagnosed, polyarticular JIA. One arm of the study used subcutaneous methotrexate (MTX) at 0.5 mg/kg/week as an initial therapy, while the other used a combined regimen of MTX, the TNF inhibitor, etanercept (ET), in addition to brief oral prednisone. As part of the TREAT trial, whole blood was collected for RNA expression studies at specific time points during the course of the first year of therapy. The TREAT study therefore represents an unprecedented opportunity to observe and describe the dynamics of therapeutic response in a chronic inflammatory disease of humans at the molecular level.

The study undertaken here was directed at determining whether mathematical methods used in social network analysis may assist in characterizing the pathologic gene expression networks that may underlie JIA, and to determine whether and how effective therapy perturbs those networks. At the same time, and equally important, we aimed to describe the alterations in a network structure that represent a failed ‘re-wiring’, that is, treatment failure. We report here the results of these analyses from longitudinal samples obtained from the TREAT study subjects.

## Methods

### Patients

Eighty-five patients were recruited into the TREAT trial between October 2007 and November 2009 [22]. All children fit international criteria for polyarticular onset JIA [23]. Sixty-two parents of these children gave written informed consent for providing these samples for translational uses. Approval for use of the specimens was given by the TREAT study oversight committee. The patients submitting samples for this current study consisted of 19 boys and 45 girls aged 2 to 14 years. Of the boys, four were rheumatoid factor (RF) positive; 17 of the girls were RF positive. At the time of enrollment (month 0), prior to treatment, 2.5 mL of blood was collected in a PAXgene tube (PreAnalytiX GmbH, Hilden, Germany). Samples were stored at –80 °C. A summary of patient characteristics is shown in Table 1. Patients were randomly assigned to one of two blinded aggressive treatment arms of the study. Arm 1 (MTX) consisted of MTX 0.5 mg/kg/week SQ (40 mg max) plus placebo etanercept SQ weekly and placebo oral prednisolone tapered to zero by 17 weeks. Arm 2 (MEP) consisted of MTX 0.5 mg/kg/week SQ plus etanercept 0.8 mg/kg/week SQ (50 mg max) and oral prednisolone (60 mg max) for 16 weeks. At 4 months, those patients not achieving an ACR pediatric 70 improvement from baseline were treated (or retreated) with open label MEP. At 6 months, those patients not achieving clinical inactive disease were changed to open label MEP treatment, if they were not already on it. Further specimens were collected at subsequent visits at 4, 6, and 12 months after enrollment. These samples are hereafter referred to as m0, m4, m6, and m12.

**Table 1:** A summary of patient characteristics

Girls (n)	45
Boys (n)	19
Age range, 2–7 years	12
Age range, 7–12 years	26
Age range, 12–17 years	26
Age, mean ± SD	10.99 ± 3.998
RF positive (n)	21
ANAs positive (n)	23

Disease activity was assessed using criteria developed by Wallace *et al.* [24]. Children were assessed to have active disease (AD) if they had synovitis in at least one joint at the time the sample was taken. For children to be assessed to have inactive disease (ID), they were required to have: zero joints with active arthritis, no fever, rash, serositis, splenomegaly or generalized lymphadenopathy attributable to JIA, no active uveitis, a normal ESR in the laboratory where tested, and a physician’s global assessment of disease activity score of 0 (0 being best possible score).

### Healthy control samples

Controls consisted of eight healthy girls and 11 healthy boys between the ages of 7 and 13 years that were recruited from the OU Children’s Physicians General Pediatrics clinic. The protocol for obtaining these specimens was approved by the University of Oklahoma IRB (#13205). Anesthesia for the phlebotomy was provided using topical lidocaine/prilocaine solution. These samples are hereafter referred to as HC.

### RNA processing

RNA was purified from whole blood PAXgene specimens using a PAXgene Blood RNA kit (Qiagen, Valencia, CA, USA) as recommended by the manufacturer, including a DNAse (Qiagen) step to remove genomic DNA. Globin transcripts, which reduce labeling efficiency of whole blood cell RNA and decrease signal-to-noise ratios on microarrays [25] were reduced using GLOBINclear-Human (Ambion, Austin, TX, USA). Final RNA preparations were suspended in RNase-free water, quantified spectrophotometrically, and analyzed for RNA integrity by capillary gel electrophoresis (Agilent 2100 Bioanalyzer; Agilent Technologies, Palo Alto, CA, USA).

### Microarray analysis

Data analysis was performed on microarray data whose preliminary results we have previously reported from the standpoint of biomarker development [26]. cRNA was produced from reverse transcribed cDNA using the Illumina® TotalPrep RNA Amplification Kit (Ambion, Inc., Austin, TX, USA), hybridized to Illumina WG-6 v3 or Illumina HT-12 v4 human whole genome microarrays, and stained according to the manufacturer’s directions. Array hybridizations were undertaken in three separate batches. The first batch consisted of the 19 healthy controls, 26 m0 samples, two m4 samples, and one m12 sample hybridized on Illumina WG-6 v3 arrays. The second batch consisted of the remaining 147 patient samples from the main study hybridized to Illumina HT-12 v4 arrays. The final independent cohort of OK samples was hybridized on Illumina WG-6 v3 slides. cRNA preparation and hybridizations of the second and third batches were carried 12 months subsequent to the analysis of the first batch. Microarray data were validated by quantitative rtPCR on an independent cohort of untreated JIA patients, as previously reported in [26].

### Analysis of differential gene expression

All statistical analyses were carried out in R [27]. To facilitate statistical analyses relative to healthy controls, it was necessary to combine data from different hybridization batches. Due to the difference in the microarrays it was necessary to create combined datasets using only those probes that were present on both array formats. Illumina probe IDs were used to identify 39,426 common probes. Datasets were variance stabilized and normalized using robust spline normalization via the *lumi* package [28, 29]. Raw and normalized data were submitted to the Gene Expression Omnibus (Series GSE55319). Batch effects were removed using the ComBat algorithm in the *sva* package [30]. Briefly, ComBat employs a parametric empirical Bayes approach to estimate scaling parameters for mean and variance of expression for each gene to compensate for systematic batch effects. The method is designed to be effective for relatively small studies and robust to outliers and has been shown to be more effective than other commonly used algorithms such as distance-weighted discrimination or surrogate variable analysis [31]. Prior to statistical analysis, non-responding probes were filtered out of the datasets using the detection *P* value provided by the Illumina quality control metrics to eliminate probes not responding at higher than background levels. Analysis of differential gene expression between patients and controls was performed by fitting a linear model to the expression data using the *limma* package [32, 33]. False discovery rate (FDR) was estimated using the method described by Benjamini and Hochberg [33]. Statistical significance of gene expression was determined at FDR ≤0.05. Validation of gene array analysis was accomplished from an independent cohort of children with JIA using real-time, quantitative rtPCR for selected genes. These data have been reported elsewhere [26].

### Dynamic gene co-expression network construction

From more than 39,000 measured genes in our JIA microarray gene expression set, we selected 2,000 genes that had the smallest *P* values (via t-test, where the *P* value is 1.75 e-47) across the patient and control groups. While this cutoff is arbitrary, it specifically selects those genes whose expression values best distinguish children with disease from healthy children. The process for constructing the gene co-expression network was as follows: (1) calculation of the correlation between each gene pair via Pearson correlation coefficients; (2) after pair-wise correlation was calculated, we defined a threshold to establish the pair-wise gene relationships. Only gene pairs that had correlation values larger than the threshold were considered as having an interaction. We chose the top 10 % of gene pairs having the highest correlation coefficients. This approach allows us to transform the continuous matrix data into discrete network data.

The rule for selecting the interactions was as follows: on the one hand, we wanted the constructed gene co-expression networks to be able to display characteristics of scale-free networks, as demonstrated by the previous work [34, 35]; on the other hand, the constructed networks were required to be connected (that is, demonstrate a path connecting each node pair). Note that this is the general method for constructing gene co-expression networks [36, 37].

**Fig. 1:**
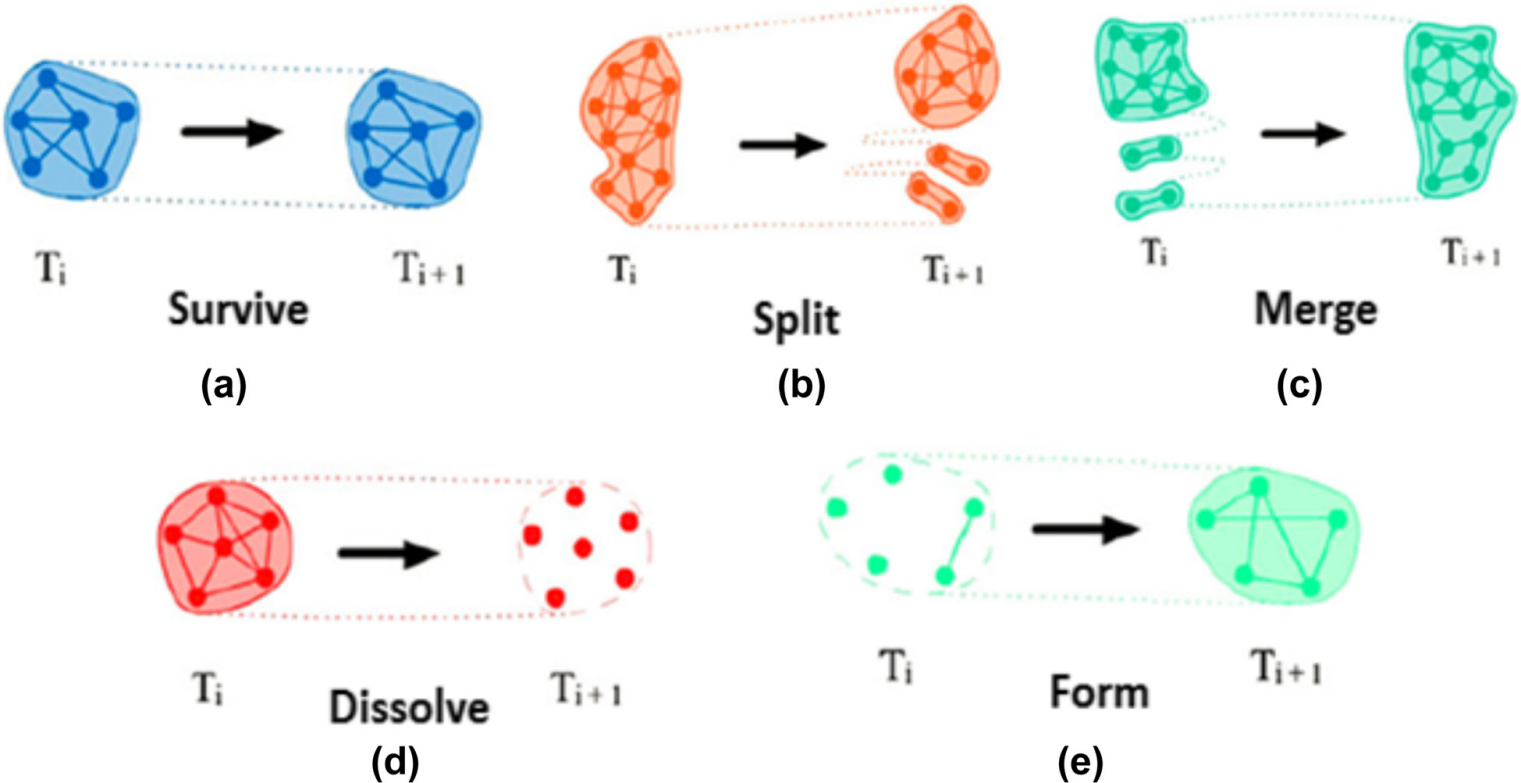
Examples of five evolutionary events: form, dissolve, survive, split, and merge are displayed. **a** Form: a community in timestamp i-th survives until the i + 1-th timestamp. **b** Split: a community in timestamp i-th splits into several communities in i + 1-th. **c** Merge: several communities in timestamp i-th timestamp merge into one community in i + 1-th timestamp. **d** Dissolve: a community in timestamp i-th disbands in the i + 1-th timestamp. **e** Form: a community does not exist in i-th timestamp appear in i + 1-th timestamp

We defined the problem of characterizing the evolutionary life of the functional modules in dynamic gene co-expression networks in the following way. At a particular timestamp *i*, we can detect a set of functional modules, denoted as ${C}^i=\left\{{C}_1^i,{C}_2^i,\dots, {C}_{k_i}^i\right\},$ from the dynamic gene co-expression network *W*^*i*^. Note that there may be overlapping between modules generated by this clustering method. We defined five different evolutionary events that are related to functional module changes: form, dissolve, continue, split, and merge (as shown in Fig. 1). These key evolutionary events cover the evolution of functional modules and can be further formulated as a set of rules which are described below.

Given a module *C*_*x*_^*i*^ from *i*-th timestamp, the metric which tracks the closest module that has the highest similarity with *C*_*x*_^*i*^ at (*i* + 1)-th timestamp is defined as:

$$ \begin{array}{c}\hfill \kern1.32em  track\left({C}_x^i,i+1\right)={C}_y^{i+1}\;iff\hfill \\ {}\hfill {C}_y^{i+1}= argma{x}_{C_z^{i+1}\in {C}^{i+1}}\left\{\frac{\left|{V}_x^i{\displaystyle \cap }{V}_z^{i+1}\right|}{max\left(\left|{V}_x^i\left|,\right|{V}_z^{i+1}\right|\right)}\right\}\ge \alpha, \hfill \end{array} $$

where *V*_*x*_^*i*^ is the set of proteins of *C*_*x*_^*i*^, and the overlap threshold α defines whether two modules are matched in a given overlap ratio, which is also used in the definitions of evolutionary events below. So this module similarity measures the optimal matching module for *C*_*x*_^*i*^ at (i + 1)-th timestamp. If none of the modules in *C*^*i+*1^ has an overlap ratio larger than α, then return ∅ (∅ denotes an empty matching result).

#### Form

**Fig. 2:**
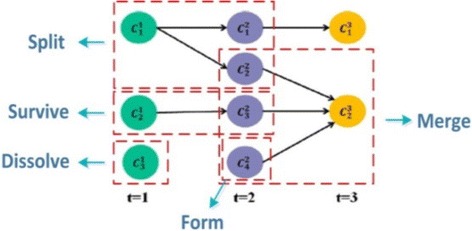
An example of functional module evolution over three timestamps. Five evolutionary events including form, dissolve, survive, split, and merge are demonstrated

A particular functional module *C*_*x*_^*i*^ is marked as having formed if it did not exist in the previous timestamp. To be more specific, a form indicates that it is the first time a set of genes for proteins that are grouped together by common functionality; some examples of first formed modules *C*_1_^1^, *C*_2_^1^, and *C*_4_^2^ are shown in Fig. 2. Thus, module *C*_*x*_^*i*^ is formed in the *i*-th timestamp iff:

$$ track\left({C}_x^i,i-1\right)=\phi $$

#### Dissolve

A dissolve occurs for a particular functional module *C*_*x*_^*i*^ if no similar module exists in the next timestamp. Specifically, a dissolve indicates that it is the last time a set of proteins are grouped together to perform some function, and an example of module *C*_3_^1^ is shown in Fig. 2. Formally, a module *C*_*x*_^*i*^ in the *i*-th timestamp is defined as dissolve iff:

$$ track\left({C}_x^i,i-1\right)=\phi $$

#### Continue

Modules are designated as continuing if there is a particular functional module *C*_*y*_^*i*^ + 1 detected in timestamp *i* + 1 that is close to a module *C*_*x*_^*i*^ in the previous timestamp *i*-th. We then say *C*_*y*_^*i*^ + 1 is the continuation of *C*_*x*_^*i*^ in the next timestamp. It can also be considered as a module which continues its existence in the consecutive timestamps. In Fig. 2, module *C*_3_^2^ is the continuation of module *C*_2_^1^. Formally, a module *C*_*x*_^*i*^ in the *i*-th timestamp continues its existence to the (*i* + 1)-th timestamp iff:

$$ \exists {C}_y^{i+1}\in {C}^{i+1}\; track\left({C}_x^i,i+1\right)={C}_y^{i+1} $$

#### Split

If a particular functional module *C*_*x*_^*i*^ in *i*-th timestamp is matched to a set of modules in the coming i + 1-th timestamp then we say *C*_*x*_^*i*^ is split. For example, in Fig. 2, module *C*_1_^1^ is split into two modules -- *C*_1_^2^ and *C*_2_^2^ in the next timestamp. Formally, a module *C*_*x*_^*i*^ in the *i*-th timestamp is split into a set of modules *C*_1_^*i* + 1^, *C*_2_^*i* + 1^, …, *C*_*k*_^*i* + 1^ in the (*i* + 1)-th timestamp iff:

$$ \begin{array}{c}\hfill \exists {C}_{*}^{i+1}=\left\{{C}_1^{i+1},{C}_2^{i+1},\dots, {C}_k^{i+1}\right\}\subseteq {C}^{i+1}:\hfill \\ {}\hfill \forall {C}_y^{i+1}\in {C}_{*}^{i+1}:\frac{\left|{V}_x^i{\displaystyle \cap }{V}_y^{i+1}\right|}{\left|{V}_y^{i+1}\right|}\ge \alpha \hfill \end{array} $$

#### Merge

If a particular functional module *C*_*x*_^*i* + 1^ in (*i* + 1)-th timestamp is matched to a set of modules C_*_^i^ = {*C*_1_^*i*^, *C*_2_^*i*^, …, *C*_*k*_^*i*^} in the previous *i*-th timestamp then we say *C*_*x*_^*i* + 1^ is merged from *C*_1_^*i*^, *C*_2_^1^, …, *C*_*k*_^*i*^, and C_*_^i^ ⊆ *C*^*i*^. For example, in Fig. 2, module *C*_2_^3^ is merged from three modules *C*_2_^2^, *C*_3_^2^, and *C*_4_^2^ in the previous timestamp. Formally, a set of modules ${C}_1^i,C{}_2^1,\dots, {C}_k^1$ in the *i*-th timestamp is merged into a module *C*_*x*_^*i* + 1^ in the (*i* + 1)-th timestamp iff:

$$ \forall {C}_y^i\in {C}_{*}^1:\frac{\left|{V}_y^i{\displaystyle \cap }{V}_x^{i+1}\right|}{\left|{V}_y^i\right|}\ge \alpha . $$

### Functional module strength progression analysis

Besides detecting the evolutionary events of functional modules, tracking the temporal progression of these functional modules may also provide significant insights into the disease or mechanisms of therapeutic response. In the field of data mining, community (in our case, module) analysis in dynamic networks has recently attracted attention [38–40]. However, the module information provided by current approaches is limited; these existing methods cannot provide a complete view of how gene modules evolve through the entirety of a specific observation period.

**Fig. 3:**
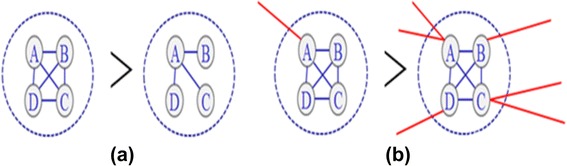
An example of module strength estimation. **a** More interactions inside a module make it stronger. **b** The more interactions a module with the outside, the less strong it is

Aiming to track the temporal changes of gene modules at each timestamp, we propose a novel measure called module strength analysis. We propose that a functional module demonstrates high strength if it has more internal interactions connecting the molecules inside it than the external interactions connecting it to the rest of the network. Dense internal interactions and weak external interactions to the outside suggest that this functional module will have low risk of change (current gene(s) leaving or/and new gene(s) joining), which may influence the biological functions of this module. For example, if one gene cluster has high strength in a particular stage, we believe that this gene cluster is active in this stage; otherwise, the function of this gene cluster may be depressed. This assumption is demonstrated in Fig. 3.

In the biological domain, interactions between genes change gradually in dynamic gene co-expression networks. Thus the strength of gene modules also changes. For example, it has been reported that the expression of key genes change [41] as the cancer progresses. In such cases, the corresponding gene modules’ strength also changes. Discovering the strengths of gene modules throughout a specific disease progression (or therapeutic response, as in our case) may provide useful clues to the underlying biology. For our specific disease, JIA, if a gene module is strong at diagnosis, such a module may be monitored through treatment to determine whether module strengthening or weakening is associated with disease response or refractoriness.

To precisely estimate each functional module’s strength, we considered the following: (1) when calculating the strength of a specific module corresponding to a particular time point, we should also consider the historical networks, that is, those in the previous time stamp. Biological networks usually evolve gradually and may be influenced by the fact that biological samples (including those used here) may contain mixed populations of cells. At the same time, experimental design or measurement errors also contribute to the ‘noisiness’ of gene expression data generated from hybridization-based gene microarrays [42]. Thus, community strength determined from only a single source, sample, or time point is difficult to assess precisely. In addition, we believe that, in the setting of chronic illness, community strength will change gradually instead of dramatically. Because response to therapy in juvenile arthritis occurs gradually (over weeks or months), we expect a certain level of temporal smoothness between module strengths in successive snapshots; such an assumption is both biologically plausible and allows us to filter out ‘noise’ that is inherent in whole blood microarray data. Therefore, we used both the current and previous biological networks to calculate the temporal community strength. (2) Since it is hard to determine whether a module is strong without comparing it with other modules, we normalized the strengths of all modules. Finally (3), the overall strength of all modules at each timestamp was also estimated.

In our previous work on social networks [43], we proposed an integrated optimization framework that conducts community (module) strength detection across snapshots by taking all the requirements mentioned above into consideration. To be more specific, we first identify the temporal functional modules at each timestamp via a clustering method, and all functional modules ascertained by this method are then collected into a candidate set. Next, the strength of each detected module corresponding to each specific snapshot is calculated through solving an objective function. Using this approach, we estimated each functional module’s strength over the time course of treatment response in JIA.

### Analysis of dynamic network changes

From the whole blood gene expression profiles from children with JIA enrolled in the NIH-funded study, we undertook two separate analyses, one of which was strategy-based and the other of which was time-based.

For the strategy-based analysis, we adopted four different operational occurrences over the course of the TREAT study. The first stage represents the baseline and denotes the gene expression profiles of patients at diagnosis. The second stage contains the data at the stage of the trial where patients begin or restart use the open label drug (patients at this stage may have been on either MTX or MTX + etanercept – see Methods for the design of the TREAT trial). These patients have had an unsatisfactory course and were therefore switched to (or restarted on) the more aggressive arm of the protocol. Alternatively, in the second stage, children have had a satisfactory response, and therefore continued to use the blinded drug. Finally, the third stage represents the gene profiles of patients who had achieved inactive disease as defined by the clinical trial protocol. Thus, the four stages can be summarized as:

1. Baseline
2.
  - Unsatisfactory initial response – Patients switched to open label drug (MTX, etanercept, and oral corticosteroids). Patients who were on this arm of the protocol had treatment re-initiated with the same drugs and tapering oral corticosteroids.
  - Satisfactory response – These patients were maintained on blinded study drug.
3. Achieved inactive disease status.

**Fig. 4:**
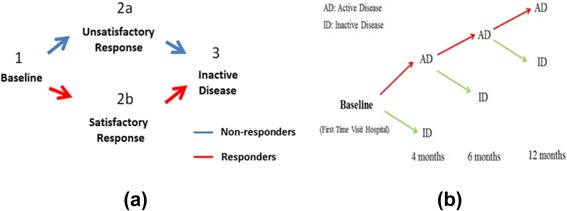
Structures of the strategy-based and time-based analyses. Panel (**a**) shows the two strategy-based analysis: non-responders are the patients follow the strategies through baseline- > unsatisfactory response- > inactive disease (blue arrow). Responders are the patients follow the strategies through baseline- > satisfactory- > inactive disease. Panel (**b**) shows the time-based analysis: active disease (AD) cases represent the patients with disease symptoms through baseline to the 12 months and inactive disease (ID) cases represent patients without disease symptoms at those specific timestamps. Analyses are based on 28 available baseline samples, 47 samples in Group 2a, 20 available samples in Group 2b, and 52 samples from patients who achieved inactive disease at 12 months

To track the differences between open labeled and blinded drug, we separated these four stages into two scenarios: ‘non-responders’ (that is, those children who were changed to open label drug) and ‘responders’ (that is, those children who were maintained on blinded drug). For the first case, we analyzed those patients whose unsatisfactory response to the blinded drug prompted a switch to open label drug and were started on the more aggressive side of the protocol. Therefore, these patients followed the stages (1)-(2a)-(3), as shown in Fig. 4a and were designated ‘non-responders’. In the second case, we analyzed expression profiles of those patients who had a satisfactory response to the blinded drug(s) and continued on those same drug(s). These patients followed the stages (1)-(2b)-(3), and were designated ‘responders’ (Fig. 4a). Note, therefore, that this analysis excludes those children who still had active disease at 12 months, as the endpoint in this analysis was the attainment of ID, whether on the original therapy or open labeled drug.

In addition to the strategy-based analysis, we also undertook a time-based analysis, comparing treatment phenotypes (active disease vs. inactive disease) at each of the time points at which samples were available. In this case, the first stage still denotes the baseline, that is, newly diagnosed, untreated disease. The second stage describes patients at 4 months, while the third and fourth stages describe patients at 6 and 12 months, respectively. Figure 4b shows the strategy for the time-based analysis.

## Results

### Temporal characteristic of the dynamic networks

**Fig. 5:**
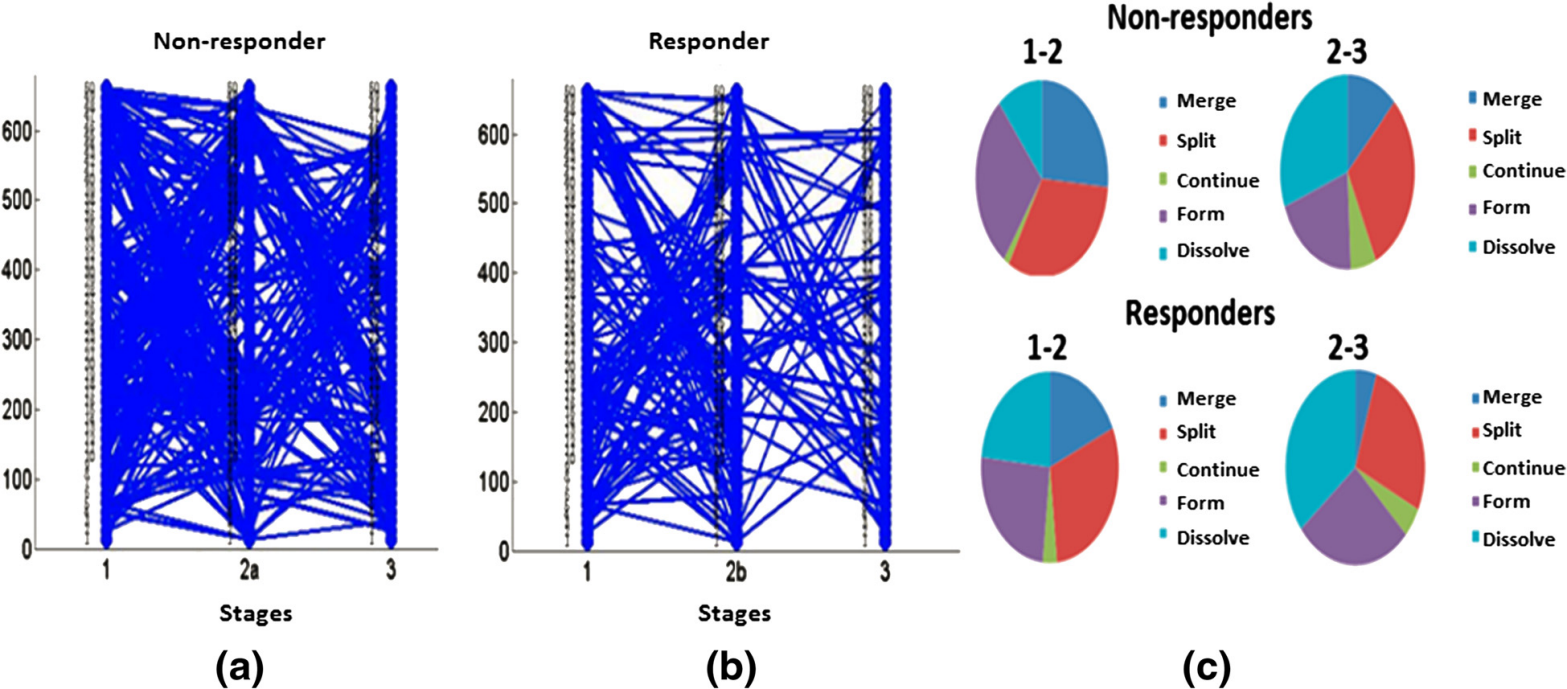
Temporal characteristics of time-based networks showing active disease (AD) patients (top panel, A) and inactive disease (ID) patients (lower panel, B), where the y-axis denotes the value of the corresponding topological measure and the x-axis denotes the four different timestamps (baseline, 4 months, 6 months, and 12 months). **a** Change of the average between-ness measure throughout the timestamps. **b** Change of the average clustering coefficient measure throughout the timestamps. **c** Change of the average closeness measure through the timestamps

Before undertaking dynamic analysis of gene expression networks, we first asked whether we could detect dynamic changes from stage to stage in the time-based networks. To accomplish this task, we used four different topological measurement methods: average gene between-ness centrality [44], average gene closeness centrality [45], and average gene correlation coefficients [44]. Although each of these methods uses different formulas, all of them are designed to measure a node’s relative importance within a network. The results of these four measurements for AD and ID patients are shown in Fig. 5, which demonstrates the dynamic change for each of these measures. In addition, each of the measures from the same group (that is, AD or ID) shows a similar pattern of change. That is, the temporal characteristic of AD patients dramatically decreases from 0 months to 4 months, and then gradually increases to 12 months. The temporal characteristic of ID patients also dramatically decreased from 0 months to 4 months (similar to the AD patients), then gradually increases to 6 months, and finally decreases to 12 months. Note that all patients showed the same patterns at 4 months regardless of whether their illness was successfully treated and they continued to experience active disease throughout the 12 months they were followed. Thus, we conclude that these properties are not characteristics of either successful or unsuccessful therapy in JIA despite the subtle differences in the patterns seen between the two groups.

### Evolutionary events analysis of functional modules

Based on different stages’ gene networks, we were able to elucidate corresponding temporal functional modules in treated JIA. We next developed a module-status-based framework for characterizing the evolution of these functional gene modules. We characterized the transformation of these modules by defining and identifying specific critical module evolutionary events using our previously published method for detecting such events [46]. The evolutionary pattern of functional modules can be represented as a sequence of key evolutionary events (changes) in consecutive timestamps. We used these evolutionary events to compute and characterize novel behavior-oriented measures, which offer insights into the characterization of dynamic behavior of functional modules.

First, we detected evolutionary events based on the strategy-based analysis. We used the Non-negative Matrix Factorization (NMF) [47] clustering method to detect functional modules at each stage of treatment response. In order to assess clustering at each timestamp, it is necessary to determine a module number into which the genes are partitioned. We preset this module number at each stage as 50, and using this setting, the gene number of each gene module contains 30 to 180 genes, within the range of previous studies [48]. Thus, at each specific stage, we selected 50 functional modules and tracked the evolutionary events among them.

**Fig. 6:**
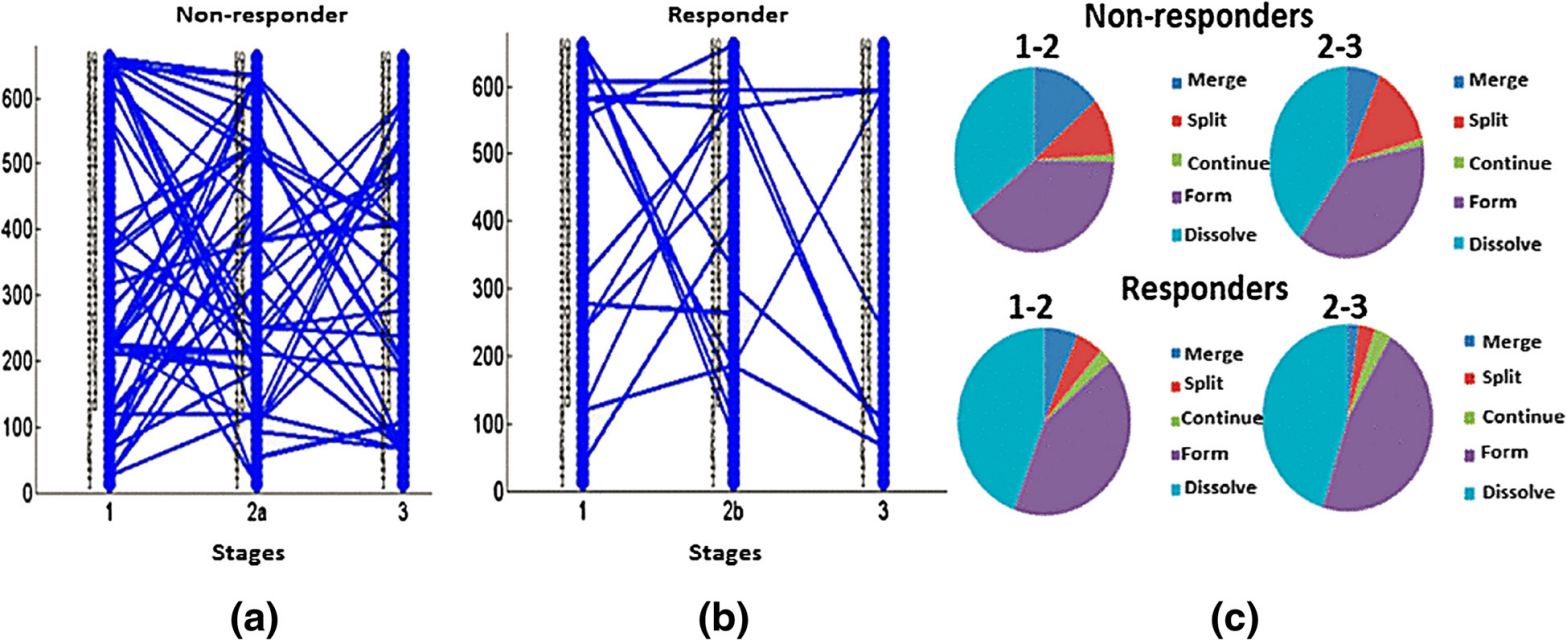
Evolutionary events detected from strategy-based analysis with the threshold set to 0.12. Stages refer to those shown in Fig. 4a. The 50 gene clusters are aligned vertically at each timestamp. In this figure: **a** The evolutionary events detected in non-responders where the functional modules detected at stages 1, 2a, and 3 interact with each other; **b** The evolutionary events detected in responders where the functional module detected at stages 1, 2b, and 3 interact with each other. **c** Pie graphs of evolutionary event calculation for each non-responders (top) and responders (bottom), where the percent of five evolutionary events at a certain time gap are shown. Note the relative paucity of ‘continue’ events in either responders or non-responders, indicating that treatment is associated with significant re-ordering of network structures regardless of therapeutic response

**Fig. 7:**
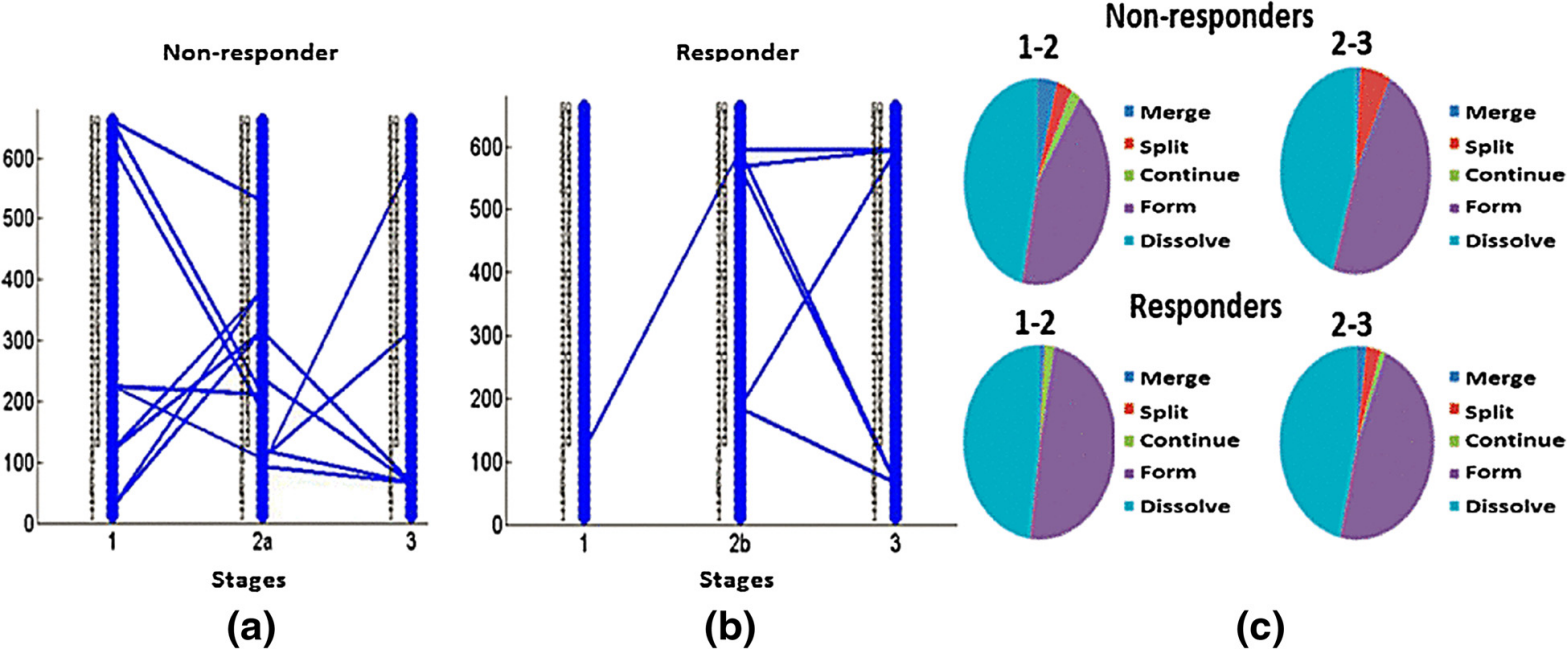
Evolutionary events detected from strategy-based analysis when the threshold is set to 0.16. The 50 gene clusters are aligned vertically at each timestamp. **a** The evolutionary events detected in non-responders where the functional module detected at stages 1, 2a, and 3 interact with each other; **b** The evolutionary events detected in responders where the functional module detected at stages 1, 2b, and 3 interact with each other. **c** Pie graphs of evolutionary event calculation of both non-responders and responders, where the percent of five evolutionary events at a certain time gap are shown. Note again the paucity of ‘continue’ events as well as the greater number of ‘dissolve’ events in the responders

**Fig. 8:**
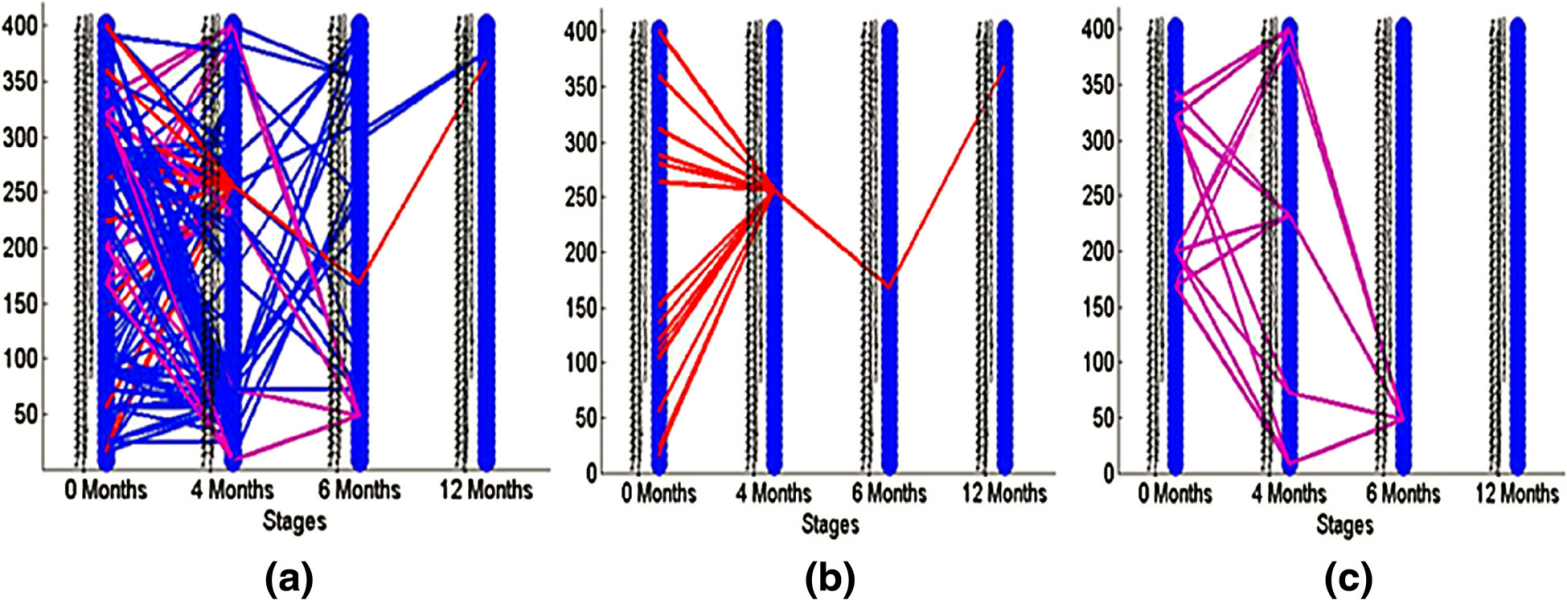
Evolutionary events detected from strategy-based analysis when threshold is set to 0.2. The 50 gene clusters are aligned vertically at each timestamp. **a** The evolutionary events detected in non-responders where the functional module detected at stages 1, 2a, and 3 interact with each other; **b** The evolutionary events detected in responders where the functional module detected at stages 1, 2b, and 3 interact with each other. **c** Pie graphs of evolutionary event calculation for both non-responders and responders, where the percent of five evolutionary events at a certain time gap are shown. Note that, even in this more stringent analysis, non-responders have more merge, split, and continue events than responders during the first 4 months of therapy (that is, the time period between stage 1 (baseline) and stage 2a or 2b)

As mentioned above, it is necessary to establish a threshold to define the matching of evolutionary events; the higher this threshold, the greater the confidence in the result. We varied the threshold from 0.12 to 0.2 with increments of 0.04. For each threshold, we constructed plots and calculated the numbers of evolutionary events, as shown in Figs. 6, 7, and 8. In Fig. 6a, we show evolutionary events detected in non-responders where the functional modules detected at stages 1, 2a, and 3, as described in Fig. 4, interact with each other. This is the pattern shown in children who initially had a poor response and were changed to open label drug. In contrast, Fig. 6b shows the evolutionary events detected in responders, where the functional modules detected at stages 1, 2b, and 3 of Fig. 4 interact with each other. Note that responders had noticeably fewer modules persist from baseline to stage 2b (where they were assessed to have had a satisfactory response) and from 2b to the achievement of inactive disease status (Stage 3). This pattern becomes clearer in Fig. 7. Figure 6c shows pie graphs of showing the types of evolutionary events for non-responders (top) and responders (bottom), where the percentage of the five types of evolutionary events at a certain time frame is shown. Note the relative paucity of ‘continue’ events in either responders or non-responders, indicating that treatment is associated with significant re-ordering of network structures regardless of therapeutic response even in non-responders. Figures 7 and 8 show that as the threshold increases, more form and dissolve events are found. Because the threshold is used to measure the similarity between the modules, the higher this threshold, the more interactions between modules that will be filtered out (with the remaining modules having a higher statistical probability of being biologically valid). Moreover, non-responders (that is, those who failed their initial treatment and were placed on open label drug) have more close connections (that is, more merge, split, continue events are found) than responders (that is, those who had had a satisfactory response and therefore remained on their blinded drug(s). In other words, the gene modules change in non-responders more gradually than responders, consistent with the clinical observation that the non-responders do not have a satisfactory therapeutic response until being switched to (or restarted on) open label drug. Moreover, in the group who failed initial therapy and were moved to the open label arm of the protocol (that is, those patients passing through stages 1, 2a, and 3), there were more detected events (merge, split, continue) than seen for those patients with a satisfactory response (stages 1, 2b, and 3), as noted above. This was predictable, as the therapeutic course is more complex in those patients who achieved ID status only after switching to open label drug, even if this was the arm of the protocol to which they were initially randomized. Finally, we note that there were relatively few ‘continue’ events detected at any of the thresholds used for analysis. This finding suggests that, whether successful or nor, treatment for JIA results in extensive re-wiring of pathology-associated gene expression networks.

**Fig. 9:**
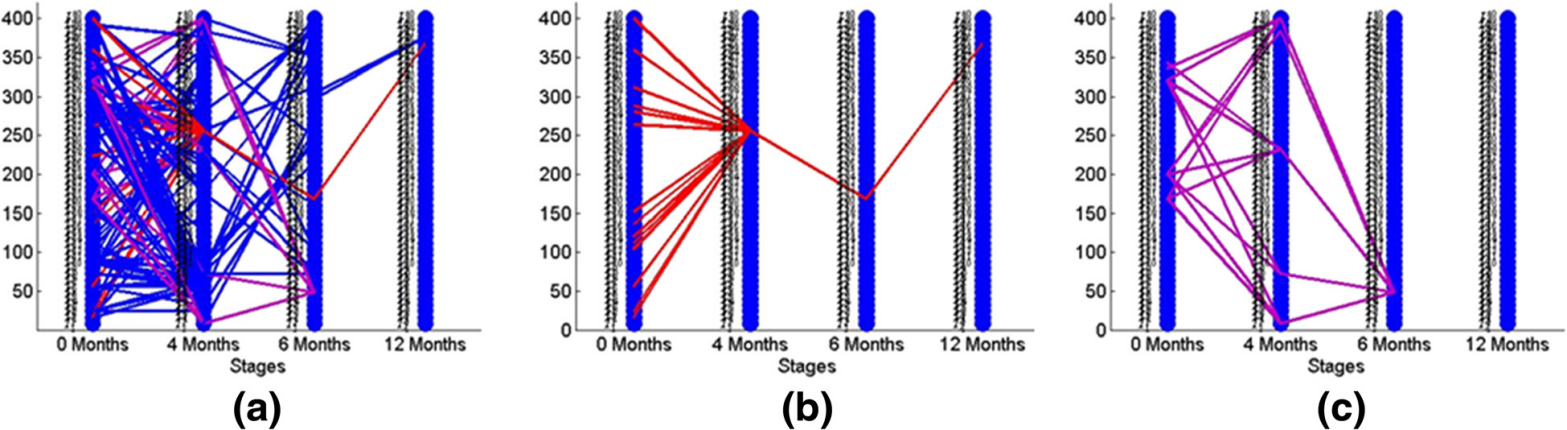
Evolutionary events detected from time-based analysis of patients with persistently active JIA. The 50 gene clusters are aligned vertically at each timestamp. **a** Red lines represent a set of gene modules which merge into a single module at the 4-month time point and persist through the 12 months of the trial. **b** Purple lines demonstrate a set of modules which split into another set of gene modules at 4 months, and finally merge into one module in the third timestamp

We next applied the proposed evolutionary event detection method on patients with persistently active disease in the time-based analysis dataset. Similar to the strategy-based analysis, we selected 50 modules at each timestamp, and then analyzed the evolutionary events between the modules. Figure 9a demonstrates the evolutionary events detected when the threshold was set as 0.14. Two interesting module evolution patterns emerged, the first of which represents a set of modules which merge to a single module at the second timestamp (4 months into therapy) and remains consistent until final timestamp (1 year into the trial, shown as Fig. 9b). The second pattern represents as a set of modules which split into another set of gene modules in the 4 months, merge into a single module in the 6 months (shown as Fig. 9c) and which finally disappears at 12 months.

### Result of module strength progression

**Fig. 10:**
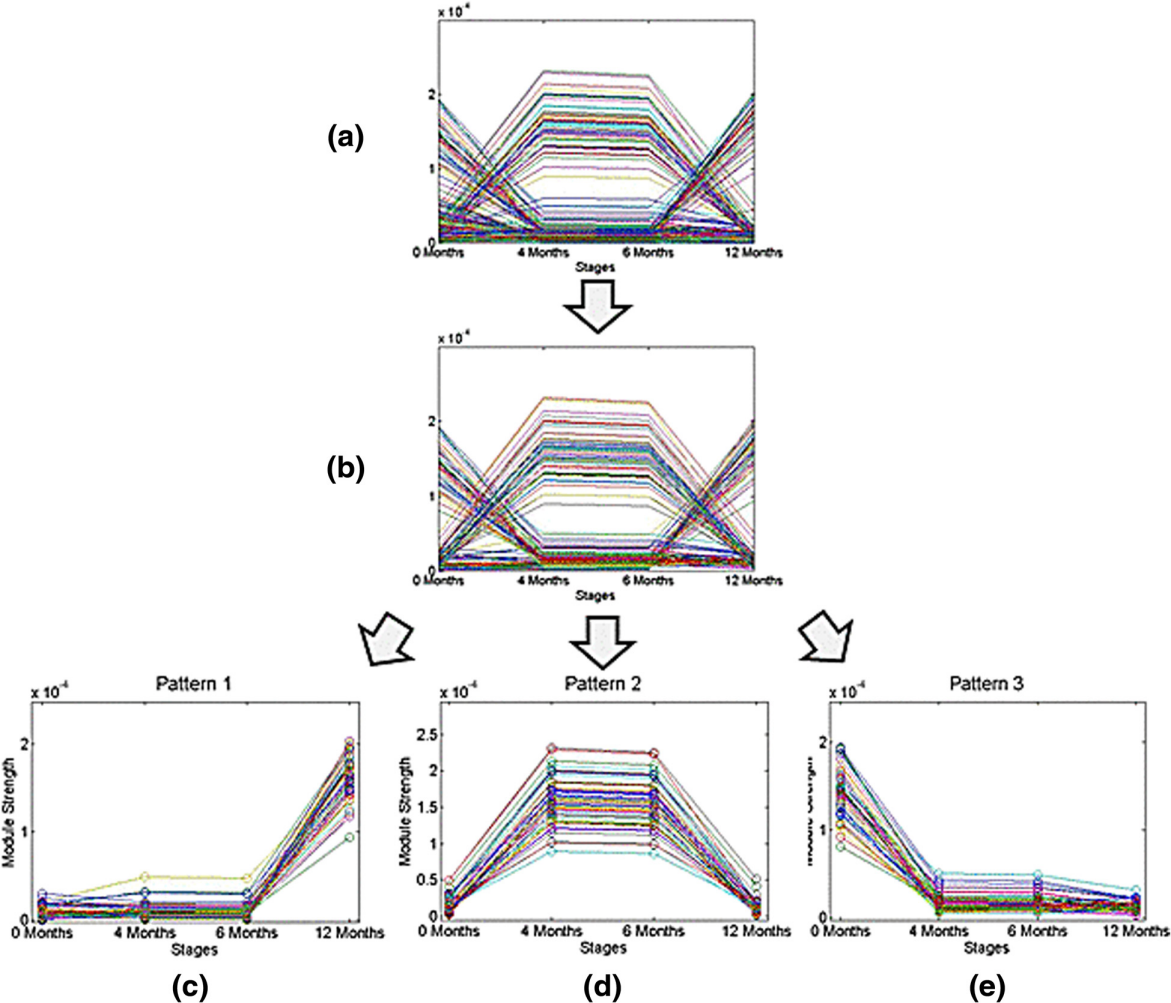
Module strength patterns detected in patients with persistently active disease patients from the time based analysis, where each curve represents the change of a functional module throughout the timestamps. The y-axis denotes the module strength and the x-axis denotes the four different timestamps. **a** Strength curves of functional modules related to each time point; **b** Strength curves of functional modules related to each time point after removing modules whose patterns did not specifically correlate with phenotype (in this case, active disease); panels **c**-**e** thus show the patterns which are superimposed upon one-another in (**b**) as (**c**) the first pattern of the strength curves, whose strength is low at the first three timestamps, and dramatically increases at the last timestamp. **d** Second pattern of the strength curves, whose strength changes as a hill shape, increasing and then decreasing over time; **e** A third pattern of the strength curves, whose strength is high at the first timestamp but dramatically drops at the following timestamps

Using the time-based approach, we selected 50 functional modules from each stage, which resulted in a total of 192 modules that we inserted into our observed module set after removing duplicate modules. The number of genes in each of these modules was in the range of 50 to 180. Using the module strength progression method described in the Methods section and Fig. 3, we observed specific patterns related to each stage of treatment response of active disease (AD) patients as show in Fig. 10a. We observed that some modules’ strength curves are relatively flat compared with the others. Because the strength characteristics of these modules are unchanged over the course of therapy, regardless of whether it was successful, we assume that these modules are not critical in determining therapeutic response and eliminated them from further analysis (Fig. 10b). From the remaining modules, we find that the gene modules generally follow three patterns as shown in Fig. 10c, d, and e.

**Fig. 11:**
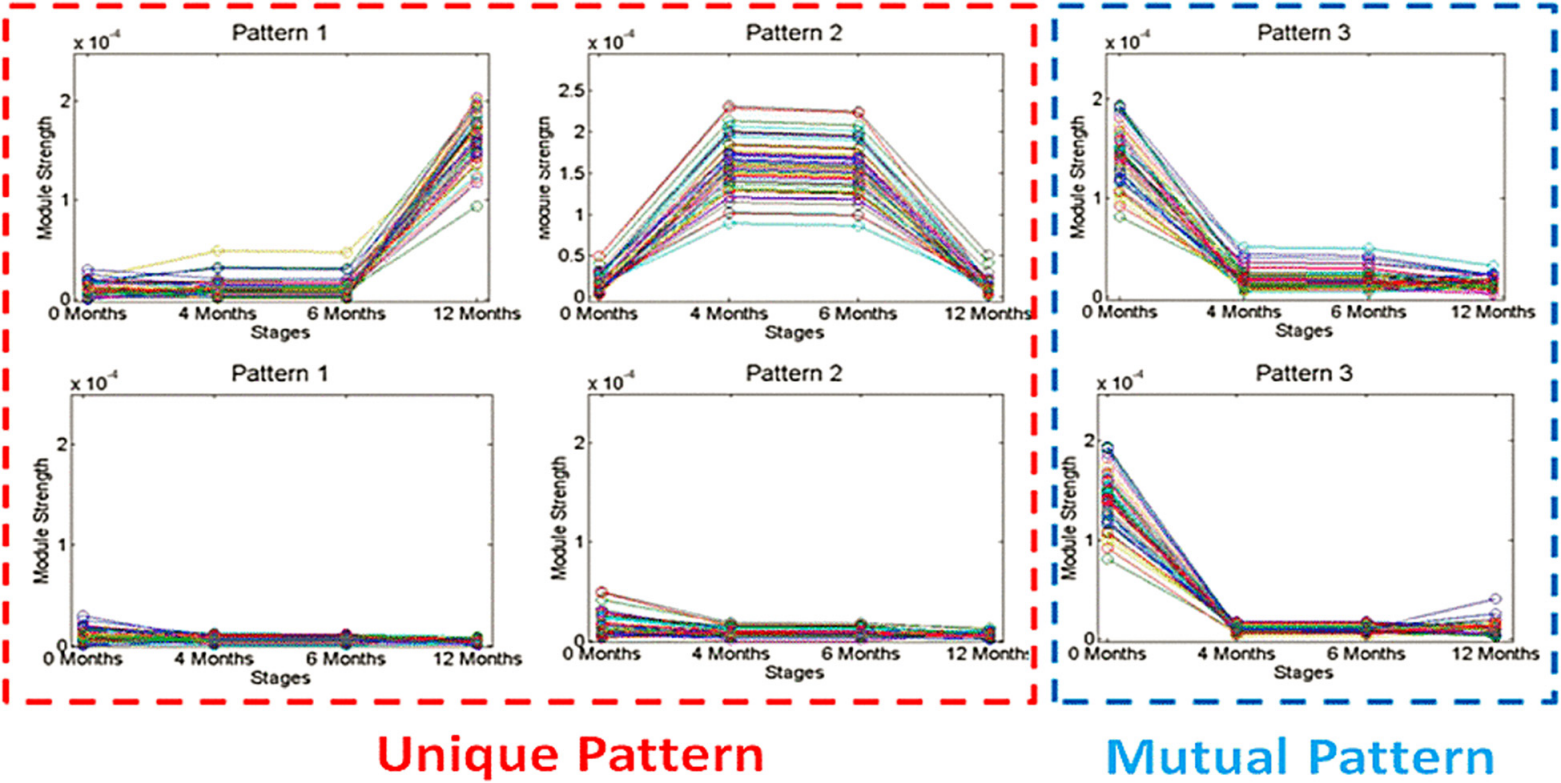
Two patterns are found through the comparing between AD (top) and ID (bottom) groups in time-sequence analysis of network cohesiveness. The two patterns on the left (Patterns 1 and 2) differ between the AD and ID patients. In Pattern 1, there is a set of modules that increases in cohesiveness in the 6- to 12-month time period only in the AD patients. Note in Pattern 2 the increase in module cohesiveness in the AD patients over the baseline to 4-month time period. This properties of this same set of modules remains unchanged in the ID group. These findings are consistent with clinical data from the TREAT trial demonstrating the importance of the first 4 to 6 months of therapy in determining longer-term response. The final pattern (Pattern 3) shares similar evolutionary features between the two groups

Based on the module strength results, we next compared these three patterns with their corresponding strength evolutionary patterns in the time-based analysis. In other words, we sought to determine whether these modules would have significantly different representations in AD and ID groups. Results are shown in in Fig. 11. Two patterns, those designated ‘c’ and ‘d’ in Fig. 10 and Pattern 1 and Pattern 2 in Fig. 11, perform differently across the AD and ID groups. This suggests that these communities represent specific biological functions which are related to the AD group. On the other hand, Pattern 3 demonstrates nearly identical trends in both AD and ID groups. This suggests that it is very likely that these communities represent some mutual functions shared between AD and ID groups.

To gather a better biological understanding of these patterns, we randomly sampled 15 modules (approximately 15 % to 20 % of each pattern) from each pattern, and submitted them to functional analysis using the Database for Annotation, Visualization and Integrated Discovery (DAVID) Software (v6.7), a National Institute of Allergy and Infectious Disease-supported analysis tool that allows functional analysis of large genomic datasets [49]. By using random sampling, the analysis results are more likely to be representative and free from bias and clustering errors, as this approach gives each cluster of a population an equal chance of being selected.

As Fig. 11 shows, two functional annotations are depressed early in therapy but strengthen between 6 and 12 months in patients with persistently active disease. For Pattern 2, we also found two functional annotations which show significant differences different across the pattern. These are the modules that show significant strengthening between 0 and 4 months only in those patients with persistently active disease. These functional groups are GO:0006811 (ion transport) and GO:0045165 (cell fate commitment). These processes are important in both leukocyte activation and terminal differentiation to effector cells.

**Fig. 12:**
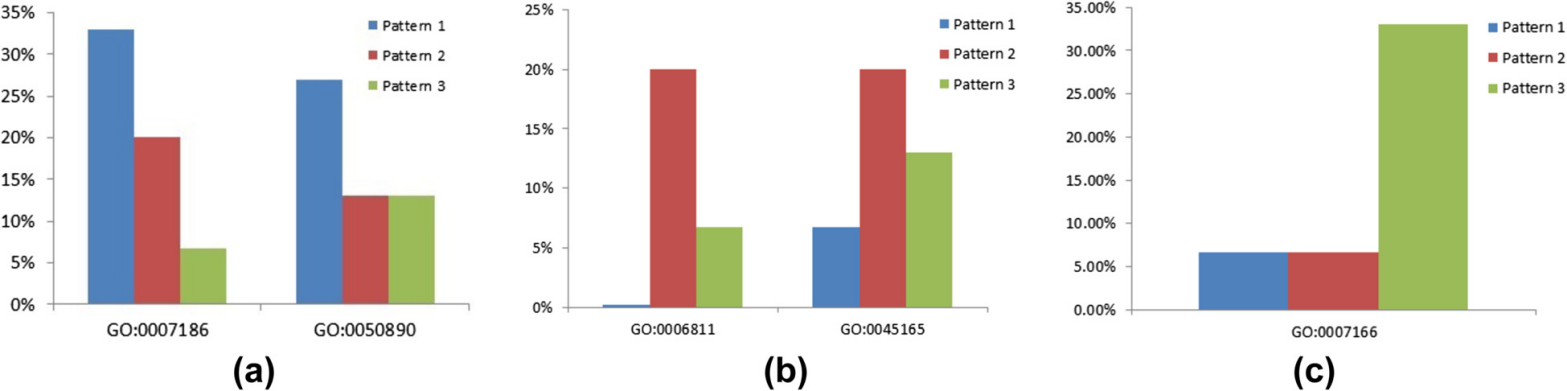
Enrichment of functional annotations of genes represented in Fig. 10c-e. **a** Two different gene functional annotations, GO:0007186, G-protein coupled receptor signaling, and GO: 0050890 (cognitive functioning) are enriched for Pattern 1 as shown by the blue bar. **b** Two different represented gene annotations (GO:0006811, cell fate commitment and GO:0045165, cell fate commitment) are enriched in Pattern 2. On both annotations, the percent taken in Pattern 2 (red bar) is clearly larger than Pattern 1 (blue bar) and Pattern 3 (green bar). **c** One different represented gene annotation (GO: 0007166, cell surface receptor linked signal transduction) is enriched in Pattern 3. The percent taken in Pattern 3 (green bar) is clearly large than Pattern 1 (blue bar) and Pattern 2 (red bar). For all annotations, the percent of genes identified by the functional annotations is shown on the y-axis

For Pattern 1, we found two functional annotations that showed statistically significant enrichment when compared with the other patterns: GO:0007186 (G-protein coupled receptor protein signaling pathway) and GO:0050890 (cognition), where the frequencies are shown in Fig. 12a. The latter result was unexpected, although complex relations between immune system activation and cognitive functioning have long been suspected in adult rheumatoid arthritis [50].

As Fig. 11 shows, these two functional annotations are depressed in the first three stages, but after stage 4 (inactive stage) is reached they start to be active.

For Pattern 2, we also found two functional annotations which show significant differences different across the pattern. These functional groups are GO:0006811 (ion transport) and GO:0045165 (cell fate commitment); the frequencies are shown in Fig. 12b.

For Pattern 3, we found that the gene annotation GO: 0007166 (cell surface receptor linked signal transduction), which shows remarkable different across patterns, where the frequencies are shown in Fig. 12c. As the result shows, this annotation is only active at the first stage, but after that it begins to depress. Thus, it is possible that the drug has an influence on this biological function.

In the time-based analysis, we have detected 50 functional modules from each stage of AD group, and there are 188 modules are inserted into our observed module set after removing the duplicate modules.

For these detected modules, we have also made the gene cluster strength analysis. First of all, we have learned three cluster strength evolutionary patterns from AD group which look similarly to the patterns we found from the strategy-based analysis. The reason why the detected patterns are similar is that the patients used for constructing the strategy-based and the AD group in the time-based analysis are actually highly overlapping.

To better demonstrate the functions behind these patterns, we further look down into the gene annotation of these patterns, respectively. We calculate the frequency of the corresponding annotation terms of all the modules belonging to this pattern as Table 2 shows. As we can see that, the frequencies we found are also close with the statistical results of the corresponding patterns detected in the strategy-based analysis.

**Table 2:** Annotation frequency analysis for patterns derived from the active disease (AD) group in time-based analysis

	GO Term	Frequency
Pattern 1	GO:0007186 – G-protein coupled receptor signaling pathway	32.40 %
	GO:0050890 – cognition	32.40 %
Pattern 2	GO:0006811 – ion transport	10.70 %
	GO:0045165 – cell fate commitment	14.20 %
Pattern 3	GO:0007166 – cell surface receptor linked signal transduction	30.00 %

## Discussion

One of the fundamental findings of modern biology has been the discovery that gene expression is tightly coordinated across the genome [51]. Furthermore, gene expression occurs in such a way as to form complex networks [52] that display a high degree of cohesiveness and robustness [53]. These networks permit cells to function properly in the setting of multiple perturbations in the surrounding milieu [4, 6] and thus are likely to be essential to survival of both single and multi-cellular organisms. The findings from basic biology have led to the hypothesis that human illnesses emerge because of disturbances in these complex cellular networks [11, 12], and there is clinical and experimental evidence to support this hypothesis [54, 55]. Indeed, even ‘simple’ Mendelian traits appear to demonstrate complex alterations and network ‘rewiring’ that was previously unsuspected [56]. In this paper, we demonstrate that medical intervention itself is associated with complex alterations in gene expression networks, and that different patterns of rewiring are associated with efficacy and degree of treatment response.

Juvenile idiopathic arthritis (JIA), the illness studied here, has long been assumed to be a complex trait characterized by gene-environment interactions [15], and is one of the most common chronic illnesses in children [57, 58]. The illness is characterized by inflammation and synovial hypertrophy in affected joints, and, prior to the availability of effective therapies, frequently resulted in permanent functional impairment or disability. Therapy with methotrexate and biological inhibitors of tumor necrosis factor (TNF) is now common practice and provides most children with prolonged periods without disease symptoms [59, 60]. Long categorized as an autoimmune disease, it is now known that this illness probably emerges from complex interactions between the adaptive and innate immune systems and includes specific aberrations in neutrophil function [19, 61].

In this study, we used mathematical approaches previously used to analyze social networks [43] to probe the biological basis of treatment response/non-response in JIA. In this paper, we identified disease-associated networks, as we have previously described [43]. Treatment was associated with rapid re-ordering of these networks, even in patients whose therapeutic response was inadequate. Regardless of the stringency of the selection process used map network evolution events (as described in Fig. 1), very few networks persisted unchanged after the first 4 months of therapy. This finding is consistent with the clinical data from the TREAT study subjects, which demonstrated that the first 4 months of treatment are crucial in determining therapeutic response (57), and is also consistent with additional analyses we have undertaken with the TREAT study gene expression data (unpublished data). Furthermore, non-responders demonstrated merge, split, and continue events more than responders. Finally, when we examined network cohesiveness through the lens of specific network properties, we found distinct differences between children with favorable and unfavorable treatment responses (Fig. 11). Once again, many of these differences could be observed through network strength evolution over the first 4 months of therapy (Fig. 11, Pattern 2).

While there have been many efforts to define and categorize the composition of gene co-expression networks in human disease, including JIA [21], our previous work with social networks has shown that such networks also have specific properties (for example, module strength) that may change over time even when the specific components of the network remain little altered. By examining both network composition and network properties, we demonstrate the plausibility of gaining additional insights into therapeutic response that would not be available by limiting the examination to individual components of any given network. Furthermore, the networks identified via these approaches also reflected biological plausibility, as shown in functional analysis of randomly-selected networks (Fig. 12). For example, GO:0007166 (cell surface receptor linked signal transduction) ontologies link critical leukocyte activation processes into defined networks whose properties changed over the course of therapy in this study.

There are several limits to these data that must be acknowledged. The first in the unique nature of the TREAT study subjects, which also means the absence of a comparable patient group to independently corroborate our specific findings. The TREAT study was a once-in-a-generation clinical trial, with a design quite different from any treatment approach that might be used in typical clinical practice. In addition, the TREAT study subjects differed a bit from what might be seen in a routine cross-section of children with JIA, with a skewing of the TREAT subjects toward children with more severe disease. It would be impossible, even within well-developed pediatric research networks like the Children’s Arthritis and Rheumatology Research Alliance, to find comparable patients on whom to validate these findings. We should point out, however, that we have corroborated findings from the TREAT whole blood gene expression data on an independent cohort of children with untreated JIA, as reported in [26]. In the absence of a validation study for the TREAT trial itself, this this the highest level of independent corroboration available. Given that the analysis performed here relied on the statistical methods and approaches used by Jiang and Sawle [26] in the earlier paper and was corroborated independently in at least the untreated patients, there is reason to have confidence that these analyses reflect actual biologic events. This confidence is increased insofar as the data reported here are corroborated by recently published clinical findings from the TREAT study [62].

The other major limitation of these data is the inherent noisiness of whole blood gene expression data. Peripheral blood is composed of multiple different cell types and subtypes. Although Roadmap Epigenomics has shown that there are overlaps in the transcriptomes of peripheral blood cells, each of the specific cells and cell subtypes has characteristic transcripts that define the functional differences between those cells. Furthermore, many potentially important cells may circulate in low abundance, and network rewiring associated with transcriptional changes in such low abundance cells would be lost in the background ‘noise’ that would emerge from cells of higher abundance. We have found, for example, that about 56 % of the differentially expressed transcripts in the TREAT study subjects are expressed by neutrophils, the most abundant cell in the peripheral blood (unpublished observation). While we note that there were no significant changes in the composition of peripheral blood cells as measured in routine clinical analyses in the TREAT study subjects over time, we have avoided over-emphasis on specific gene networks or modules and roles they might play in determining therapeutic response, other than the random selection of specific modules for functional analysis as shown in Figs. 12. We have demonstrate that many of the interesting patterns (for example, module cohesion, Fig. 11) are associated with plausible physiologic processes as assessed by gene ontology analysis (for example, GO:0007186, G-protein signaling pathways).

## Conclusions

Treatment response in JIA can be analyzed through the lens of evolving gene expression networks. We demonstrate that treatment is associated with significant re-ordering of gene expression networks and with multiple different patterns of network/module evolution. We believe that these preliminary studies provide a framework for similar approaches and analyses which, when applied to data from purified, pathologically-relevant cells, will provide unprecedented insight into the biology of therapeutic response in this common childhood disease.
